# Re-analysis of ventilator-free days (VFD) in acute respiratory distress syndrome (ARDS) studies

**DOI:** 10.1186/s13063-023-07190-7

**Published:** 2023-03-13

**Authors:** Rejina Mariam Verghis, Cliona McDowell, Bronagh Blackwood, Bohee Lee, Daniel F. McAuley, Mike Clarke

**Affiliations:** 1 The Wellcome-Wolfson Institute for Experimental Medicine, School of Medicine, Dentistry and Biomedical Sciences, Queen’s University, Belfast, UK; 2 GlaxoSmithKline, London, UK; 3 Northern Ireland Clinical Trials Unit, Belfast, UK; 4 Centre for Population Health Sciences, Usher Institute, University of Edinburgh, Edinburgh, UK; 5 Royal Victoria Hospital, Belfast Health and Social Care Trust, Belfast, UK; 6 Centre of Public Health, Institute of Clinical Science, Queen’s University, Belfast, UK

**Keywords:** Clinical trials, Critical care, Ventilator-free days, Acute respiratory distress syndrome, Hurdle mode

## Abstract

**Background:**

Over recent decades, improvements in healthcare have reduced mortality and morbidity rates in many conditions. This has resulted, in part, from the identification of effective interventions in randomised trials, and in conducting such trials, a composite outcome measure (COM) with multiple components will increase event rates, which allows study completion with a smaller sample size. In critical care research, the COM “ventilator-free days” (VFD) combines mortality and duration of mechanical ventilation (MV) into a single continuous measure, which can be analysed in a variety of ways. This study investigates the usefulness of Poisson and two-part Poisson models compared to *t*-distribution for the analysis of VFD.

**Methods:**

Data from four studies (*ALbuterol for the Treatment of ALI (ALTA)*, *Early vs. Delayed Enteral Nutrition (EDEN)*, *Hydroxymethylglutaryl-CoA reductase inhibition with simvastatin in Acute Lung Injury (ALI) to reduce pulmonary dysfunction (HARP-2)*, *Statins for Acutely Injured Lungs from Sepsis (SAILS)*) were used for analysis, with the VFD results summarised using mean, standard deviation (SD), median, interquartile range (25th and 75th percentiles) and minimum and maximum values. The statistical analyses that are compared used the* t*-test, Poisson, zero-inflated Poisson (ZIP) and two-part Logit-Poisson hurdle models. The analyses were exploratory in nature, and the significance level for differences in the estimates was set to 0.05.

**Results:**

In HARP-2, which compared simvastatin and placebo, the mean (SD) VFD for all patients was 12.0 (10.2), but this mean value did not represent the data distribution as it falls in a zone between two peaks, with the lowest frequency of occurrence. The mean (SD) VFD after excluding patients who died before day 28 and patients who did not achieve unassisted breathing were 15.9 (8.7) and 18.2 (6.6), respectively. The mean difference (95% CI) between the two groups was 1.1 (95% CI: 0.7 to 2.8; *p* = 0.20) based on an independent *t*-test. However, when the two-part hurdle model was used, the simvastatin arm had a significantly higher number of non-zero values compared to the placebo group, which indicated that more patients were alive and free of mechanical ventilation in the simvastatin group. Similarly, in ALTA, this model found that significantly more patients were alive and free of MV in the control group. In EDEN and SAILS, there was no significant difference between the control and intervention groups.

**Conclusion:**

Our analyses show that the *t*-test and Poisson model are not appropriate for bi-modal data (such as VFD) where there is a large number of zero events. The two-part hurdle model was the most promising approach. There is a need for future research to investigate other analysis techniques, such as two-part quantile regression and to determine the impact on sample size requirements for comparative effectiveness trials.

**Supplementary Information:**

The online version contains supplementary material available at 10.1186/s13063-023-07190-7.

## Introduction

Improvements in healthcare have resulted in people living longer, and patients today have a better prognosis than even a decade ago because of lower mortality and morbidity rates. This has arisen, in part from the identification of effective interventions in randomised trials, but the decline in event rates implies that smaller differences (effect sizes) should now be expected between the groups in comparative trials. To show statistically significant smaller effect sizes, larger sample sizes, more sites for recruitment, more research staff, more regulatory requirements and usually a longer recruitment period [1]. All these factors increase the costs of research [1]. A composite outcome measure (COM) that combines two or more outcome measures can result in higher event rates and improve statistical efficiency, allowing study completion with a smaller sample size.

COMs can be classified into three main types [2]: (i) an outcome derived from a variety of component variables, (ii) occurrence of any one of the component events within a specified period and (iii) time to first occurring event within a specified period. In healthcare research, the idea of “free day” was initially proposed in 1992 [3, 4], with “free days” being a composite of survival and being “free” from receiving a resource such as organ support or ICU admission within a specified period. ICU-free days, hospital-free days and organ failure-free days are a few examples of free days.

In critical care, ventilator-free day (VFD) is generally defined as the number of days the patient was alive and free of mechanical ventilation (MV). It combines mortality and duration of MV into a single continuous measure. In the case of 28-day VFD, a patient is given a value of 0 if they die before day 28 or are still receiving MV at day 28. If, for example, the patient achieves unassisted breathing and remains ventilator-free at day 10 and alive at day 28, they are given a value of 18. VFD penalises mortality by giving the worst value of 0 if the patient dies at any time in the 28 days, which makes VFD a better outcome compared to analysing the duration of MV or duration in MV only in survivors.

In studies of patients with acute respiratory distress syndrome (ARDS), interventions are often designed to optimise respiratory parameters with the goal of improving ventilation and thus reducing time on MV. Reducing the duration of MV lowers the risk of ventilator-associated harms, length of stay in ICU and hospital and ultimately death. Thus, a COM, such as VFD, can be patient-centred and economically meaningful.

Despite its relevance in ARDS studies, VFD poses several methodological challenges. The first issue is the relative importance of components in the VFD. Mortality is a critical event, and prolonged MV is not as critical as death. As noted above, a patient who dies between 0 and 28 days is given a VFD value of 0, and a patient requiring prolonged ventilation for more than 28 days is also given this value of 0, and this is comparable to considering death as a censoring event in survival analysis, and in this scenario, it can lead to misleading results [3]. The second issue is related to the distribution of the VFD. VFD combine three mutually exclusive patient groups: those who (i) die before day 28, (ii) require prolonged MV and (iii) achieve unassisted breathing before day 28. Patients in groups (i) and (ii) receive a zero value, and those in group (iii) receive a non-zero value resulting in multiple peaks in a frequency plot, with one peak at 0 days and another in the twenties. This presents methodologic challenges in analysing and interpreting the data. The third issue is the presence of excessive “zeros” which is a separate methodological issue. These “zeros” are generated by two mutually exclusive processes “prolonged ventilation” and “death”. Patients in the former “zero” group will have had no days free of MV in the 28 days, but those in the latter did not become ventilator-free because of the competing event death, i.e. not all “0” imply “zero MV free days”. In relation to resource use, if the patient dies on day 8, the duration of ventilation is 8 days, and associated costs are for those 8 days, and for the patients who were on the ventilator for 28 days, the associated costs are higher compared to patients who die by day 8. The fourth issue is related to the type of component variables. Mortality is a binary variable (dead/alive), and duration of MV is a continuous variable (ranging from 0 to 28). Both mortality and duration of ventilation can be expressed as 0 [3], which indicates that the estimate of VFD may not reveal the mortality rate or duration of MV at the trial level unless they are reported separately. VFD is an ordinal variable, and if deaths are excluded, the VFD becomes a discrete interval variable. Bodet-Contentin and colleagues [3] presented an iso-VFD curve which showed that similar VFD estimates can be achieved for different mortality rates and duration of MV among survivors. For example, a VFD of 10 can be obtained for (i) a mortality rate of 10% and 14 days of median duration of MV among survivors and (ii) a 20% mortality rate and 10 days of median MV duration among survivors who have a VFD of 10 [3]. Therefore, the VFD is influenced by both the mortality rate and the duration of MV, and if the VFD in two groups (e.g. those receiving the interventions in a randomised trial) differ, it would not be possible to know how each of these variables is influencing the VFD value unless the components are reported separately.

Different approaches are used to analyse the duration of MV in the presence of mortality, and a single test (e.g. *t*-test, Wilcoxon rank-sum test) is widely used to analyse VFD for hypothesis testing. However, the statistical properties of VFD differ based on the number of days used to calculate the value (e.g. 28-day VFD vs. 60-day VFD) [5]. If a *t*-test is used, the survival will have a higher weight in the case of a 60-day VFD compared to a 28-day VFD because the surviving patients would have a larger value. A Wilcoxon rank-sum test, the non-parametric alternative for *t*-test, is less dependent on the normality assumption. However, the substantial number of ties due to the zeros is an issue when using a Wilcoxon test. Survival analysis is another technique used to estimate the time to successful extubation. Conventional survival analysis considers achieving unassisted breathing as the event of interest, with death and prolonged ventilation as the censoring event [6]. This type of analysis assumes that all the patients eventually achieve unassisted breathing or the event of interest, and in the event of death that assumption is violated [7]. Yehya et al. [8] proposed the use of the Fine and Gray competing risk approach to evaluate VFD, assuming achieving unassisted breathing as the event of interest and mortality as a competing event. Competing risk model is a frequently used approach when there are two or more competing events which hinder the occurrence of the event. The cumulative incidence function (CIF) is the probability of experiencing the event of interest in each time interval conditional on the patient not experiencing the event of interest or the competing event before. For example, in heart disease studies, the probability of hospitalisation due to a significant cardiac event or death, a competing risk event, is often used and is meaningful [9]. In ARDS studies, achieving unassisted breathing is a positive event and death is a negative event. Therefore, estimating the probability of achieving unassisted breathing or mortality is not very meaningful [9, 10]. If mortality is the outcome of interest, critical care studies often have short-term endpoints like 28-day mortality or hospital mortality, and survival analysis focuses on when the patient died rather than did the patient die. Based on survival analysis, survival function could appear superior for the intervention arm even though the mortality rate is identical, which confuses longer survival with better mortality, which is misleading and should be avoided [11]. The authors have previously stated that ignoring mortality when interpreting VFD can lead to misleading conclusions, but VFD is often interpreted as days free of ventilation ignoring mortality.

Poisson models are often used for positively skewed variable, like the length of hospital or ICU stay [12]. However, the presence of zeros due to death and prolonged ventilation indicates that a two-part model is more appropriate for VFD. This article investigates if Poisson and two-part Poisson models are a better fit for VFD compared to *t*-distribution.

## Method

VFD is summarised using mean, standard deviation (SD), median, interquartile range (IQR—25th and 75th percentiles) and range (minimum, maximum). The analysis compares the results based on Poisson, zero-inflated Poisson and two-part logit-Poisson hurdle with a *t*-test. We used the chi-square goodness of fit test to assess whether the expected value was significantly different from the observed value. Analyses were exploratory, and the significance level was set at 0.05. Analyses were carried out using RStudio [13], and the forest plot was created using a SAS macro by Matange [14].

### Analytic models

#### Poisson model

Denis Poisson proposed the Poisson distribution. A variable *X* with a Poisson distribution is written as:

1 $$\begin{array}{cc}{\varvec{P}}\left({\varvec{X}}={\varvec{x}}\right)=\frac{{{\varvec{\lambda}}}^{{\varvec{x}}}{{\varvec{e}}}^{-{\varvec{\lambda}}}}{{\varvec{x}}!}\end{array}$$

where, in this case, *x* would be the VFD value, ranging from 0 to 28. The Poisson distribution has only parameter *λ*, which represents the mean and variance of the distribution.

##### Zero-inflated Poisson (ZIP) model

The zero-inflation Poisson (ZIP) model was proposed by Lambert as an application to estimate the defects in manufacturing [15, 16]. Zero-inflated models are used when two kinds of zeros are thought to exist in the data, “true zeros” and “excess zeros”. Zero-inflated models have two parts, one for the count model and one for the excess zeros. In the case of VFD, zeros due to death are considered excess zeros and zeros due to prolonged mechanical ventilation is considered true zeros, “zero-free days”. The two-part ZIP model with parameters *π* and* λ* is written as:

2 $${\varvec{P}}\left({\varvec{X}}={\varvec{x}}\right)=\left\{\begin{array}{ccc} {\varvec{\pi}}+\left(1-{\varvec{\pi}}\right)\boldsymbol{*}{{\varvec{e}}}^{-{\varvec{\lambda}}} & \mathbf{i}\mathbf{f} & {\varvec{X}} = 0 \\ \left(1-{\varvec{\pi}}\right)\boldsymbol{*}\left(\frac{{{\varvec{\lambda}}}^{{\varvec{x}}}{{\varvec{e}}}^{-{\varvec{\lambda}}}}{{\varvec{x}}!}\right)& \mathbf{i}\mathbf{f}&{\varvec{X}} >0\end{array}\right.$$

where *x* would be the VFD value, *π* is the proportion of excess zeros values due to mortality and *λ* is the mean and variance.

#### Logit-poisson hurdle model

The two-part logit-Poisson hurdle model or otherwise known as zero-altered Poisson (ZAP) was introduced by Mullahy [17]. This model assumes two processes, one generating zero and another for non-zero values. The first part of the model involves a logit model for zeros vs non-zeros, and the second part is a Poisson model, with mean *λ*, for the non-zero observations. Patients crossing the “hurdle” are assigned a positive value. In the case of VFD, the hurdle is being alive and achieving unassisted breathing, and the proportion is represented by *π*. The main difference between the ZIP model and the hurdle model is that the latter does not distinguish between true zeros and excess zeros. The two-part logit-Poisson hurdle model with parameters *π* and *λ* is written as:

3 $${\varvec{P}}\left({\varvec{X}}={\varvec{x}}\right)=\left\{\begin{array}{ccc} {\varvec{\pi}}& \mathbf{i}\mathbf{f}&{\varvec{X}}=0\\ \left(1-{\varvec{\pi}}\right)\boldsymbol{*}\left(\frac{{{\varvec{\lambda}}}^{{\varvec{x}}}{{\varvec{e}}}^{-{\varvec{\lambda}}}}{{\varvec{x}}!}\right)& \mathbf{i}\mathbf{f}&{\varvec{X}}>0\end{array}\right.$$

where *x* would be the VFD value, *π* is the proportion of non-zero values and *λ* is the mean and variance.

### Data

The National Institute of Health (NIH) and National Heart Lung and Blood Institute (NHLBI) established the Acute Respiratory Distress Syndrome Network (ARDSnet) to develop an effective intervention for ARDS. Data from the HARP2 (*Hydroxymethylglutaryl-CoA reductase inhibition with simvastatin in acute lung injury to reduce pulmonary dysfunction)* trial [6] and three ARDSnet studies, *ALbuterol for the Treatment of ALI (ALTA)* [18], *Early vs Delayed Enteral Nutrition (EDEN)* [19], *Statins for Acutely Injured Lungs from Sepsis (SAILS)* [20], which reported VFD as a primary or secondary outcome, were used in this analysis.

## Results

**Fig. 1:**
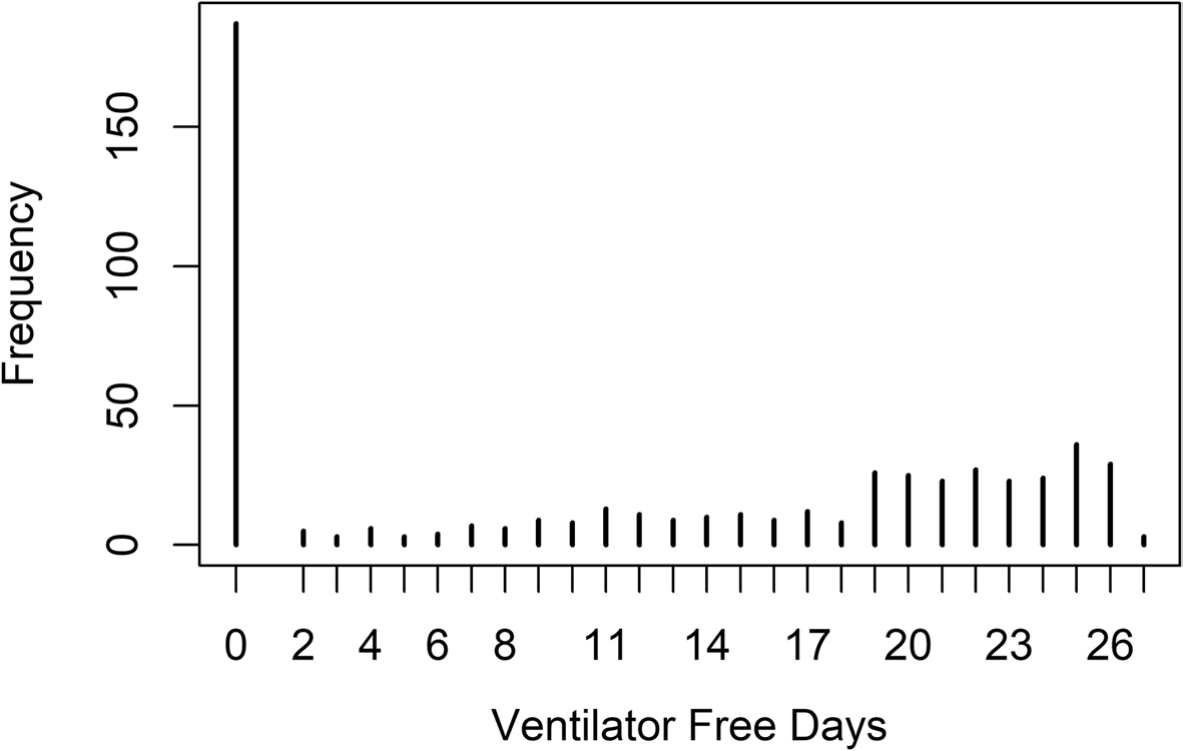
Distribution of the VFD in the HARP2 study

Figure 1 shows the distribution of the VFD for the HARP2 data. There are two peaks in the observed VFD data, one at 0 and another at 25. By day 28, there were 132 (24.6%) deaths, and 55 (10.2%) other patients had not achieved unassisted breathing [6]. The mean (SD) VFD for all patients was 12.0 (10.2). The mean (SD) VFD after excluding patients who died prior to day 28 was 15.9 (8.7). The mean (SD) after the exclusion of deceased patients and patients requiring prolonged MV was 18.2 (6.6). Table 1 shows the summary statistics for all patients, summary statistics after excluding zeros due to mortality and summary statistics for patients who achieved unassisted breathing (excludes all zeros). The change in summary statistics indicates that the average value is influenced by the zeros.

**Table 1:** Summary statistics for HARP-2, ALTA, EDEN and SAILS

Study	VFD score			VFD score for survivors^a^				VFD score for patients achieving UB^b^	
**HARP-2**	**All**	**Placebo**	**Simvastatin**	**All**	**Placebo**	**Simvastatin**	**All**	**Placebo**	**Simvastatin**
*N*	537	279	258	405	204	201	353	170	180
Mean (SD)	12.0 (10.2)	11.5 (10.4)	12.6 (9.9)	15.9 (8.70)	15.8 (9.1)	16.1 (8.3)	18.2 (6.6)	18.7 (6.4)	17.8 (6.8)
Median (IQR)	13 (0, 22)	12 (0, 22)	14 (0, 22)	19 (10 to 23)	19 (9, 23)	19 (10, 23)	20 (14, 23)	20 (14, 24)	20 (14, 23)
Min to Max	0 to 27	0 to 27	0 to 27	0 to 27	0 to 27	0 to 27	2 to 27	2 to 27	2 to 27
**ALTA**	**All**	**Placebo**	**Albuterol**	**All**	**Placebo**	**Albuterol**	**All**	**Placebo**	**Albuterol**
*N*	282	130	152	233	112	121	205	102	103
Mean (SD)	15.4 (10.6)	16.6 (10.0)	14.4 (11.1)	18.6 (8.7)	19.3 (8.1)	18.0 (9.4)	21.2 (5.8)	21.1 (5.6)	21.2 (6.0)
Median (IQR)	20 (0, 24)	21 (7, 24)	20 (0, 24.5)	22 (15 to 25)	22 (17, 25)	22 (13, 25)	23 (19, 26)	23 (17, 25)	23 (19, 25)
Min to Max	0 to 28	0 to 28	0 to 28	0 to 28	0 to 28	0 to 28	2 to 28	2 to 28	2 to 28
**EDEN**	**All**	**Full**	**Tropic**	**All**	**Full**	**Tropic**	**All**	**Full**	**Tropic**
*N*	1000	492	508	806	397	409	696	351	345
Mean (SD)	14.9 (10.8)	15.0 (10.6)	14.9 (10.9)	18.5 (8.8)	18.6 (8.5)	18.5 (9.1)	21.3 (5.5)	21 (5.5)	21.6 (5.4)
Median (IQR)	20 (0, 24)	19.5 (0, 24)	20 (0, 25)	22 (15 to 25)	22 (16 to 25)	22 (15 to 25)	23 (19, 25)	23 (18, 25)	23 (20, 25)
Min to Max	0 to 28	0 to 28	0 to 28	0 to 28	0 to 28	0 to 28	1 to 28	1 to 28	1 to 28
**SAILS**	**All**	**Placebo**	**Rosuvastatin**	**All**	**Placebo**	**Rosuvastatin**	**All**	**Placebo**	**Rosuvastatin**
*N*	745	366	379	573	285	288	515	251	264
Mean (SD)	15.1 (10.9)	15.1 (11.0)	15.1 (10.8)	19.6 (8.1)	19.4 (8.5)	19.8 (7.7)	21.8 (5.2)	21.9 (5.2)	21.6 (5.1)
Median (IQR)	20 (0, 25)	20 (0, 25)	20 (0, 25)	23 (17, 25)	23 (17, 25)	23 (17, 25)	23 (20, 26)	23 (20, 26)	24 (19.5, 25.5)
Min to max	0 to 28	0 to 28	0 to 28	0 to 28	0 to 28	0 to 28	1 to 28	2 to 28	1 to 28

^a^0 s due to 28-day mortality are excluded

^b^0 s due to 28-day mortality or prolonged ventilation are excluded

**Fig. 2:**
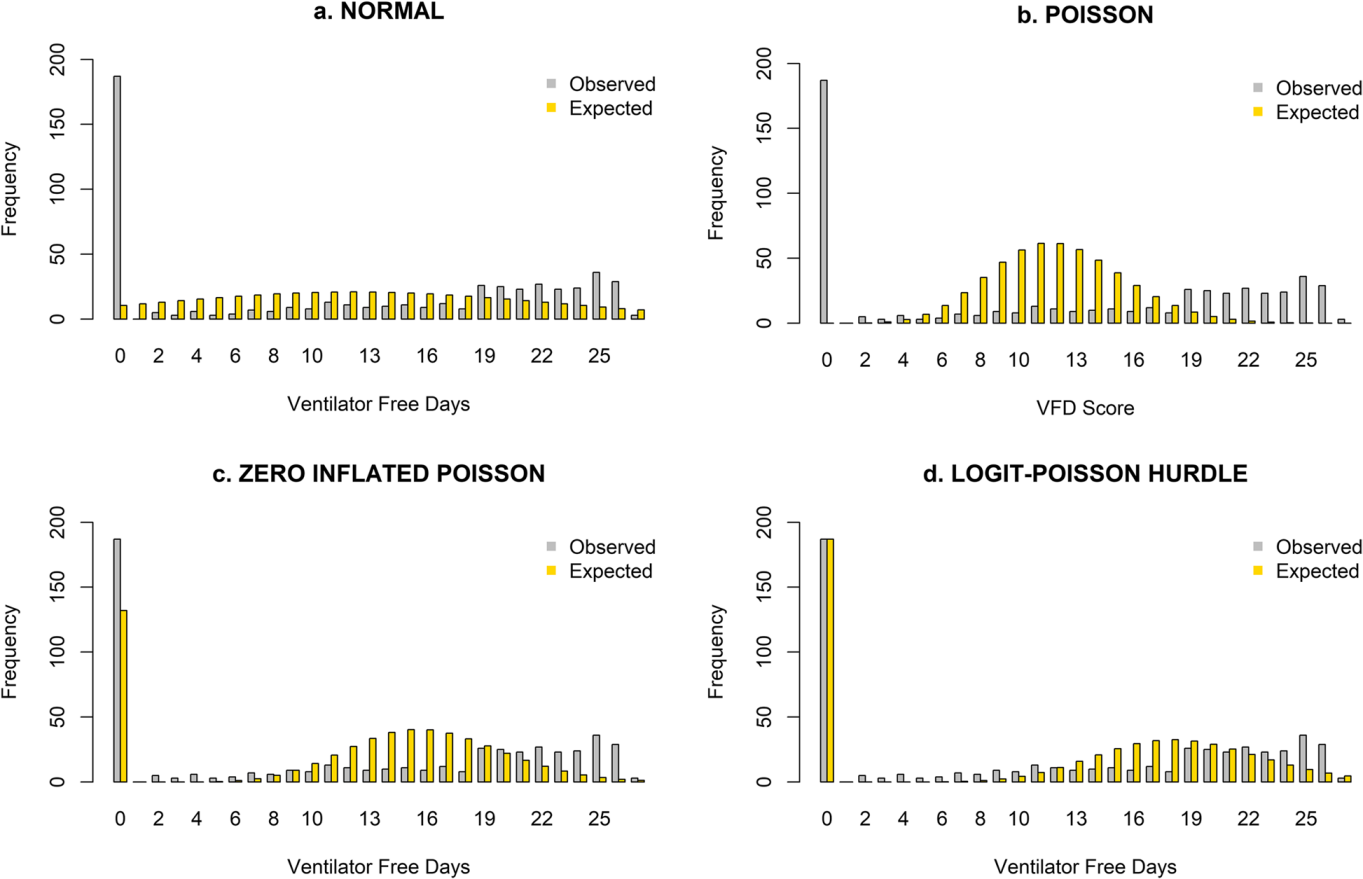
Observed and expected HARP2 VFD values for different models

Figure 2 shows the observed and expected frequencies based on all four analytical distributions for VFD in HARP2. The two-part hurdle model predicts the numbers of the zeros correctly because of the model construct: zeros versus non-zero. The non-zero values peaked around 18 as per the hurdle model, while the peak in the observed values was at 25. The expected counts of the VFD were predicted based on the parameters estimated from the data. There were 187 zeros in the data. A normal distribution estimated eleven zeros, Poisson model did not predict any zeros and the ZIP model estimated 132 zeros. The hurdle model predicted all 187 zeros because the model looks at zeros and non-zero values. In the ZIP model, the proportion of excess zeros due to mortality was predicted, and the ZIP model did not predict additional zeros. The other peak in the VFD distribution was observed at value 25. The chi-square values (Table 2) indicate that none of the models was a good fit for the VFD distribution (*p *> 0.05), but the logit-Poisson hurdle model was comparatively better.

**Table 2:** Chi-square goodness of fit statistic for HARP2, ALTA, EDEN and SAILS

*χ*^2^ statistic	HARP2	ALTA	EDEN	SAILS
OLS	292.8	168.20	618.65	486.52
Poisson	618.8	307.07	1125.11	849.82
Zero-inflated Poisson	210.9	113.53	378.58	204.05
Logit-Poisson hurdle	117.1	54.40	153.71	104.70

The ZIP model considers excess zeros due to mortality in the first part and the rest of the VFD values in the second part, while the hurdle model considers all the zeros versus non-zeros as a logistic model in the first part and the second part for non-zero values in a Poisson model.

The mortality in the placebo and simvastatin groups in HARP2 was 26.9% and 22.1%, respectively, with a difference in the mortality rate of approximately 5% favouring the simvastatin. Similarly, 12.2% and 8.1% in the placebo and simvastatin groups required MV more than 28 days, with approximately a 4% difference favouring simvastatin. After the exclusion of the zeros, the mean (SD) VFD was similar in both groups: 18.7 (6.4) for placebo and 17.8 (6.8) for simvastatin. The mean (SD) VFD was 11.5 (10.4) in the placebo group and 12.6 (9.9) in the simvastatin group; the mean difference between the groups was 1.1 (95% CI: − 0.7 to 2.8, *p* = 0.20) [13]. In the ALTA study, the mean VFD in the albuterol and placebo groups was 14.4 (11.1) and 16.6 (10.0), respectively (mean difference: − 2.2, 95% CI: − 4.7 to 0.3, *p* = 0.087). In the EDEN study, the mean VFD in the trophic-feeding group and full-feeding group was 14.9 (10.9) and 15.0 (10.6), respectively (mean difference: − 0.1, 95% CI: − 1.4 to 1.2, *p* = 0.89). In SAILS, the mean (SD) VFD in the rosuvastatin and placebo groups were 15.1 (10.8) and 15.1 (11.0), respectively (mean difference: 0.04, 95% CI: − 1.5 to 1.6, *p* = 0.96).

Additional file 1: Table S1 shows the hurdle model estimate based on data from HARP2. There are two parts to the models, the first part is the logit model for zero versus the non-zero, and the second part is the Poisson model for the non-zero values of the VFD. The logit model indicates a 48% increase in the odds of having a non-zero value of VFD if the patient is in the simvastatin group compared to the placebo group, which was statistically significant (*p* = 0.032). The count model part indicates a 4.2% decrease in VFD for patients in the simvastatin group compared to the placebo group, which was not statistically significant.

**Fig. 3:**
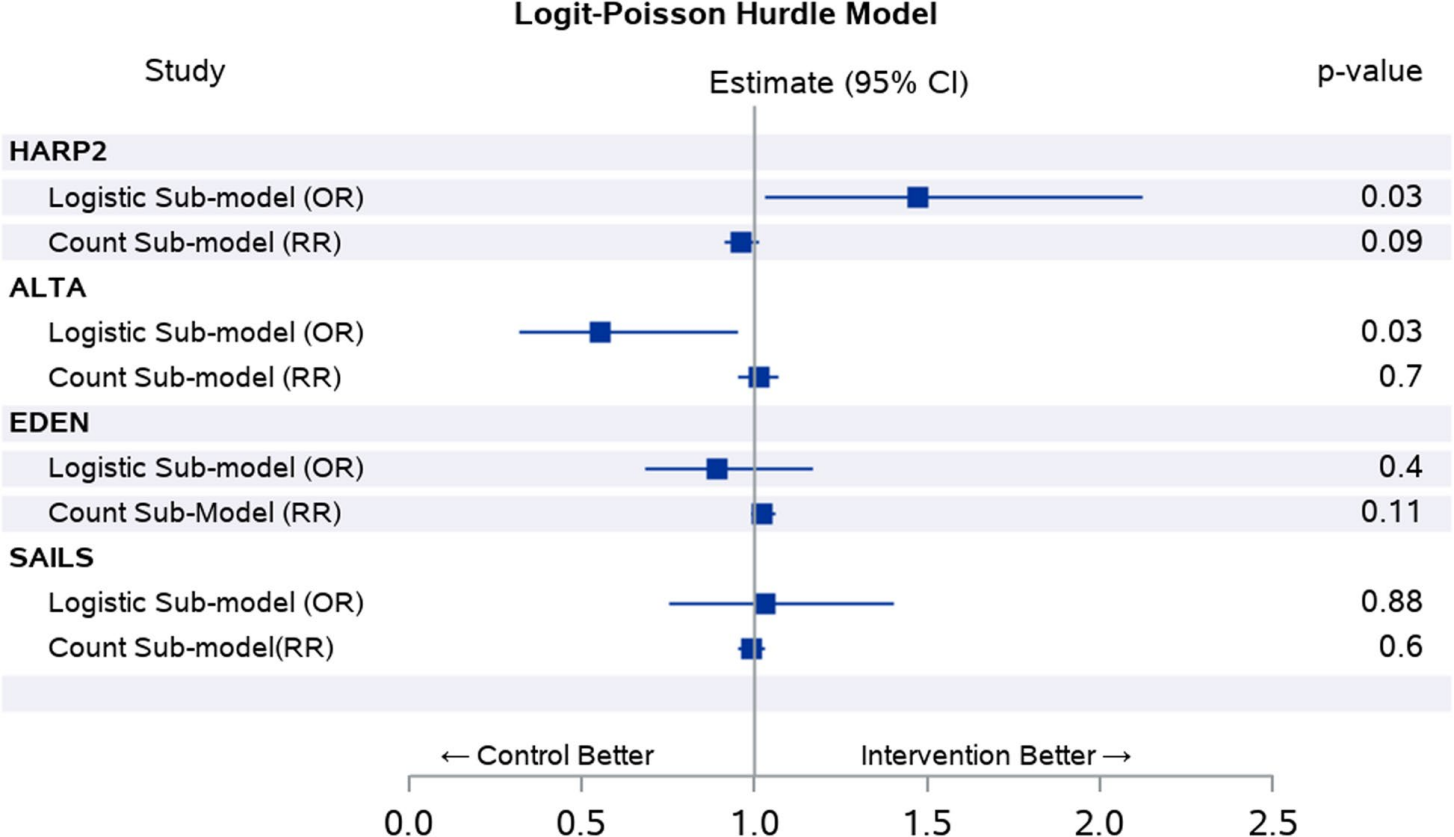
OR estimates for the logit sub-model and RR estimate for count data sub-model for ALTA, EDEN, HARP2 and SAILS

Table 3 and Fig. 3 show the odds ratio (OR) and rate ratio (RR) estimates for the logit sub-model and count data sub-model, respectively, for ALTA, EDEN, HARP2 and SAILS. This shows that, in ALTA, the number of patients achieving unassisted breathing is significantly higher in the placebo than in the albuterol group, and there was no statistically significant difference in non-zero VFD between the groups. There was no statistically significant difference between the logit sub-model and count data sub-model in EDEN and SAILS.

**Table 3:** Chi-square goodness of fit statistic for HARP2, ALTA, EDEN and SAILS

Study	Treatment group			
**HARP-2**	**Placebo**	**Simvastatin**	**Estimate (95% CI)**	***p*-value**
*N*	279	258		
Logit-Poisson hurdle model				
Logistic sub-model (*n* (%))^a^	170 (61.0%)	180 (69.8%)	1.47 (1.03 to 2.12)	0.03
Count sub-model (Mean ± SD)^b^	18.7 ± 6.4	17.8 ± 6.8	0.96 (0.91 to 1.01)	0.09
**ALTA**	**Placebo**	**Albuterol**	**Estimate (95% CI)**	***p*-value**
*N*	130	152		
Logit-Poisson hurdle model				
Logistic sub-model (*n* (%))	102 (78.5%)	103 (67.8%)	0.55 (0.32 to 0.95)	0.03
Count sub-model (mean ± SD)	21.1 ± 5.6	21.2 ± 6.0	1.01 (0.95 to 1.07)	0.70
**EDEN**	**Full**	**Tropic**	**Estimate (95% CI)**	***p*-value**
*N*	492	508		
Logit-Poisson hurdle model				
Logistic sub-model (*n* (%))	351 (71.3%)	345 (67.9%)	0.89 (0.68 to 1.17)	0.40
Count sub-model (mean ± SD)	21 ± 5.5	21.6 ± 5.4	1.02 (0.99 to 1.06)	0.11
**SAILS**	**Placebo**	**Rosuvastatin**	**Estimate (95% CI)**	***p*-value**
*N*	366	379		
Logit-Poisson hurdle model				
Logistic sub-model (*n* (%))	251 (68.6%)	264 (69.7%)	1.03 (0.75 to 1.40)	0.88
Count sub-model (mean ± SD)	21.9 ± 5.2	21.6 ± 5.1	0.99 (0.95 to 1.03)	0.60

^a^Logistic sub-model reports the *n* (%) of patients with non-zero values and odds ratio (95% CI) and *p*-value for the Logit-Poisson hurdle model

^b^Count sub-model reports the mean ± SD, rate ratio (95% CI) and *p*-value for the logit-Poisson hurdle model

## Discussion

This paper has reviewed the utility of VFD as a valid outcome measure for critical care studies and the use of* t*-test for hypothesis testing. VFD penalises the worst outcome, death, by giving death the worst value, with patients who died before day 28 having the same score (0) as those who have MV ≥ 28 days, which makes the VFD one of the few composite outcomes which efficiently captures the worst component in a composite. VFD has two peaks, one at 0 and at the twenties, because of three different patient populations: those who died before day 28, those who did not achieve unassisted breathing by day 28 and those who achieved unassisted breathing by day 28. The first two groups are patients with the worst outcomes and caused a peak at zero, while many of the third group will have been extubated during their first week of MV and provided a peak VFD in the twenties. The mean values are misleading because of bimodality, and excluding all zeros raises the mean VFD from 12.0 to 18.2 in HARP2. Similarly, if those excess zeros due to deaths are excluded, the mean VFD is 15.9. These show the importance of reporting each outcome component alongside the VFD summary to provide insight into every component in the composite outcome.

The mean (SD) VFD in all patients in the HARP2 study was 12.0 (10.2), which is in the zone between the two peaks where there is the lowest frequency of occurrence. This makes the usefulness of such mean estimates doubtful. Poisson models are frequently used to analyse the duration data which are usually positively skewed. In this paper, the Poisson model showed the worst fit for VFD and did not predict any of the zeros, which means Poisson is not appropriate for data with excess zeros. In the VFD distribution, the true zeros are for those patients who had no MV-free days or required ventilation for at least 28 days, and the excess zeros are those who died within 28 days without achieving unassisted breathing. The ZIP model was used to deal with excess zeros and uses two simultaneous equations: one for excess zeros and another for the other VFD values. The ZIP model, with treatment as the predictor value for excess zeros and other values, produced comparable results to the two-part hurdle model and its usefulness needs to be investigated further.

The two-part logit-Poisson hurdle model is like the ZIP model, with the difference being that the hurdle model does not differentiate between true zeros and excess zeros. The first part of the model involves a logit model for zeros vs non-zeros and a Poisson model, with mean* λ*, for the non-zero observations. The patients receive a non-zero value once they pass the hurdle, which in VFD is being alive and achieving unassisted breathing. In HARP2, the mean difference between the placebo and simvastatin groups was not statistically significant based on the *t*-test, but the two-part model showed statistical significance in the logistic sub-model with more patients achieving unassisted breathing in the simvastatin than the placebo group. Similarly, in the ALTA, the results based on the *t*-test were not statistically significant, but based on the two-part hurdle model, the logistic sub-model showed more patients achieving unassisted breathing in the placebo than in the albuterol group. Figure 3 shows that the count data sub-model was not significantly different across the studies and this forest plot also shows that the CI for the OR estimate is wider than the RR estimate. This study did not investigate the sample size requirement or the issue of multiple testing when a two-part logit-Poisson model is used.

The heterogeneity in the definition of VFD across trials has been reported by several authors. For example, Blackwood et al. looked at sixty-six MV trials, and twenty-five trials reported VFD as an outcome. In the 16 studies which reported a definition, start and endpoints varied [21]. Contentin and colleagues reviewed 128 reports of ICU studies that reported MV duration and/or VFD as outcomes [22]. VFD was reported in fifty-five studies of which thirty-four reported a definition, and thirteen different definitions were identified. These inconsistencies reflect a lack of standardised methods among trialists to report this outcome consistently, which can result in significant problems for systematic reviews and meta-analyses. Yehya’s paper makes the following recommendations on the definition of the VFD in randomised controlled trials [8]: (i) day of randomisation should be considered as day 0, (ii) the 28-day period for VFD calculation, (iii) extubations lasting more than 48 h should be considered successful, (iv) non-invasive ventilation and tracheotomies should not be counted in the VFD calculation and (v) all 28-day non-survivors should be given a VFD of 0 with patients censored after day 28. In contrast to this, the core outcomes of the COVENT study recommend a 60-day period for duration outcomes [23]. To assess the impact of these different durations, we are planning future research to compare two-part model results based on 28-day and 60-day VFD.

## Conclusion

VFD is a frequently reported composite outcome in critical care trials. For example, of 191 critical care COVID-19-related studies registered in ClinicalTrials.gov by July 2022, about 160 of them had VFD as an outcome. This article investigated the utility of VFD for comparing the effects of interventions in such studies and evaluated the fit of Poisson, ZIP and the logit-Poisson hurdle model compared to the *t*-test. It showed that “zeros” can cause challenges with the analyses and that a traditional mean and SD approach is not appropriate for the VFD, which implies that the *t*-test is not appropriate for hypothesis testing. The Poisson distribution had the worst fit for VFD, and the two-part logit-Poisson hurdle model was the most promising approach, which allows the analysis of zeros and non-zeros simultaneously. Future research should investigate the usefulness of other techniques, such as logit-negative binomial regression and logit-quantile regression.

## Supplementary Information

**Additional file 1: Supplemental Table S1.** HARP2 Study: Logit-Poisson Hurdle Model.

## Data Availability

This manuscript was prepared using ALTA, EDEN and SAILS research materials obtained from the NHLBI and are available in the BioLINCC repository, https://biolincc.nhlbi.nih.gov/home/. The HARP2 data are available from the study sponsor on reasonable request.
