# Supplementary material for: Re-analysis of ventilator-free days (VFD) in acute respiratory distress syndrome (ARDS) studies
Source: Trials. 2023 Mar 13;24:183. doi: 10.1186/s13063-023-07190-7 (PMC10008713; doi:10.1186/s13063-023-07190-7)
Supplement: Supplementary file 1 — Additional file 1: Supplemental Table S1. HARP2 Study: Logit-Poisson Hurdle Model. [file 13063_2023_7190_MOESM1_ESM.docx]

Supplemental Table S1. HARP2 Study: Logit-Poisson Hurdle Model

| Logit Sub- Model | | | | | |
| --- | --- | --- | --- | --- | --- |
|  | Estimate (SE) | OR | **95% CI** | | p-value |
|  |  |  | LL | UL |  |
| Intercept | 0.44 (0.12) | 1.56 | 1.23 | 1.98 | 0.0003 |
| Simvastatin | 0.39 (0.18) | 1.48 | 1.03 | 2.12 | 0.032 |
| Count Data Sub-Model | | | | | |
|  | Estimate (SE) | RR | **95% CI** | | p-value |
|  |  |  | LL | UL |  |
| Intercept | 2.93 (0.02)) | 18.81 | 18.17 | 19.47 | <0.001 |
| Simvastatin | -0.04 (0.02) | 0.96 | 0.91 | 1.01 | 0.085 |
